# Sleep Disturbances in Rats With Genetic Pre-disposition to Spike-Wave Epilepsy (WAG/Rij)

**DOI:** 10.3389/fneur.2021.766566

**Published:** 2021-11-05

**Authors:** Evgenia Sitnikova

**Affiliations:** Institute of Higher Nervous Activity and Neurophysiology of Russian Academy of Sciences (RAS), Moscow, Russia

**Keywords:** spike-wave epilepsy, genetic rat model, slow wave sleep, intermediate stage of sleep, microarousals

## Abstract

Wistar Albino Glaxo Rijswijk (WAG/Rij) rats are widely used in basic and pre-clinical studies as a valid genetic model of absence epilepsy. Adult WAG/Rij rats exhibit generalized 8–10-Hz spike-wave discharges (SWDs) in the electroencephalogram. SWDs are known to result from thalamocortical circuit dysfunction, and this implies an intimate relationship between slow-wave EEG activity, sleep spindles, and SWDs. The present mini review summarizes relevant research on sleep-related disturbances associated with spike-wave epilepsy in WAG/Rij rats in the domain of slow-wave sleep EEG and microarousals. It also discusses enhancement of the intermediate stage of sleep. In general, sleep EEG studies provide important information about epileptogenic processes related to spike-wave epilepsy.

## Introduction

Generalized spike-wave activity is a paroxysmal electroencephalographic (EEG) pattern occurring in various types of seizures, such as absence, myoclonic, atonic, tonic, and tonic–clonic seizures (1). The classical 3-Hz spike-wave discharges (SWDs) are manifestation of childhood absence epilepsy and juvenile absence epilepsy (2, 3). Some inbred rats are genetically prone to develop spontaneous generalized 8–10-Hz SWDs in their electroencephalogram (4–6). Two of these strains, Wistar Albino Glaxo Rijswijk (WAG/Rij) and Genetic Absence Epilepsy rats from Strasburg (GAERS), are considered valid genetic models of absence epilepsy (7). The present paper is focused on the experimental data obtained in WAG/Rij rats.

WAG/Rij rats are widely used in fundamental and pre-clinical research in Russia, Hungary, Turkey, Iran, Italy, Hungary, USA, UK, and others (8–10). Our institution (Institute of Higher Nervous Activity RAS, Moscow, Russia) obtained WAG/Rij rats from Radboud University Nijmegen (Netherlands) in the middle of the 90th years of the last century. Since that time, the incidence of SWDs in Moscow's colony of WAG/Rij rats slightly changed likely due to genetic drift. In 2011, we detected a relatively late debut age of spike-wave epilepsy in Moscow's population of WAG/Rij rats (7–8 months) as compared to the original population (11). In 2016, we defined female and male WAG/Rij rats without EEG seizures (12). These “non-epileptic” subjects did not express any SWDs at the age of 11–13 months, and now we breed them and select “non-epileptic” WAG/Rij substrain (NEW). Another challenging problem is substantial variation of time–frequency properties of SWDs across “epileptic” individuals. Taking into account time–frequency profile of SWDs in WAG/Rij rats, we defined fully developed (or genuine) SWDs and immature (or rudimentary) SWDs (13, 14). The EEG structure of SWDs in WAG/Rij rats and in epileptic patients is known to be similar (15). Inasmuch as here I refer to EEG seizure activity in the WAG/Rij rat model, I use the term “spike-wave epilepsy” instead of “absence epilepsy.”

It is commonly acknowledged that SWDs result from aberrant functioning of thalamocortical neuronal networks [(6, 16–18), see (19)]. As far as spike-wave epilepsy is directly linked to abnormal thalamocortical rhythmogenesis, it is considered a prototypical thalamocortical dysrhythmia (17, 20). In the last several decades, many researchers have thoroughly studied thalamocortical circuit abnormalities underlying SWDs (6, 16–18, 21–24). At the same time, thalamocortical neuronal circuitry is known to be involved in physiological slow-wave sleep oscillations, such as delta waves (22), that are critical for sleep homeostasis (25). Therefore, abnormal thalamocortical network function may underlie sleep disturbances.

Neurophysiological mechanisms of sleep regulation in epileptic subjects are known to be impaired [see (26)]. Mircea Steriade and Péter Halász epitomized this issue by the slogan “sleep and epilepsy are bedfellows (27).” In WAG/Rij rats, spike-wave epilepsy is associated with relatively mild sleep disturbances. Here I concentrate only on two of them in relation to (1) slow-wave sleep and (2) the intermediate stage of sleep. A deficiency of rapid eye movement (REM) sleep in WAG/Rij (28) is interesting, but it has not been explored so far.

## Thalamocortical Oscillations and Slow-Wave Sleep

Thalamocortical circuitry is known to produce physiological sleep oscillations such as slow-wave delta and spindles and epileptic SWDs (6, 16–19, 21–24). There is an intimate relationship between slow-wave EEG activity, sleep spindles, and SWDs (6, 16, 19, 21, 23, 29, 30). As it was shown in feline generalized penicillin epilepsy, sleep spindle could give rise to SWDs: “*transformation of spindles to spike and waves is the consequence of a single feature: increased excitability of cortical neurons to spindle-inducing thalamocortical volleys”* (29). Functional links between sleep spindles and SWDs were intensively studied using *in vivo, in vitro*, and *in silico* models [reviewed by (19, 21, 30, 31)]. In WAG/Rij rats, SWDs preferably occurred during passive wakefulness (33.1%) and light slow-wave sleep (48.4%) (32). Furthermore, “*spike-wave activity in rats is often interspersed with spindles and that it is no exception that spike-wave discharges are preceded by spindling”* (33). Sleep spindles and SWDs share a common thalamocortical mechanism but originate from different neuronal sources: sleep spindles are produced by thalamic neurons, but SWDs are produced by neocortical hyperexcitable networks (6, 19, 21, 24, 30, 34). The focal epileptic zone in WAG/Rij rats is located in the facial projection area of the somatosensory cortex, layers 5/6 [see (34)]. There is a common circadian mechanism governing SWDs and slow-wave sleep [reviewed in (35)].

**Figure 1:**
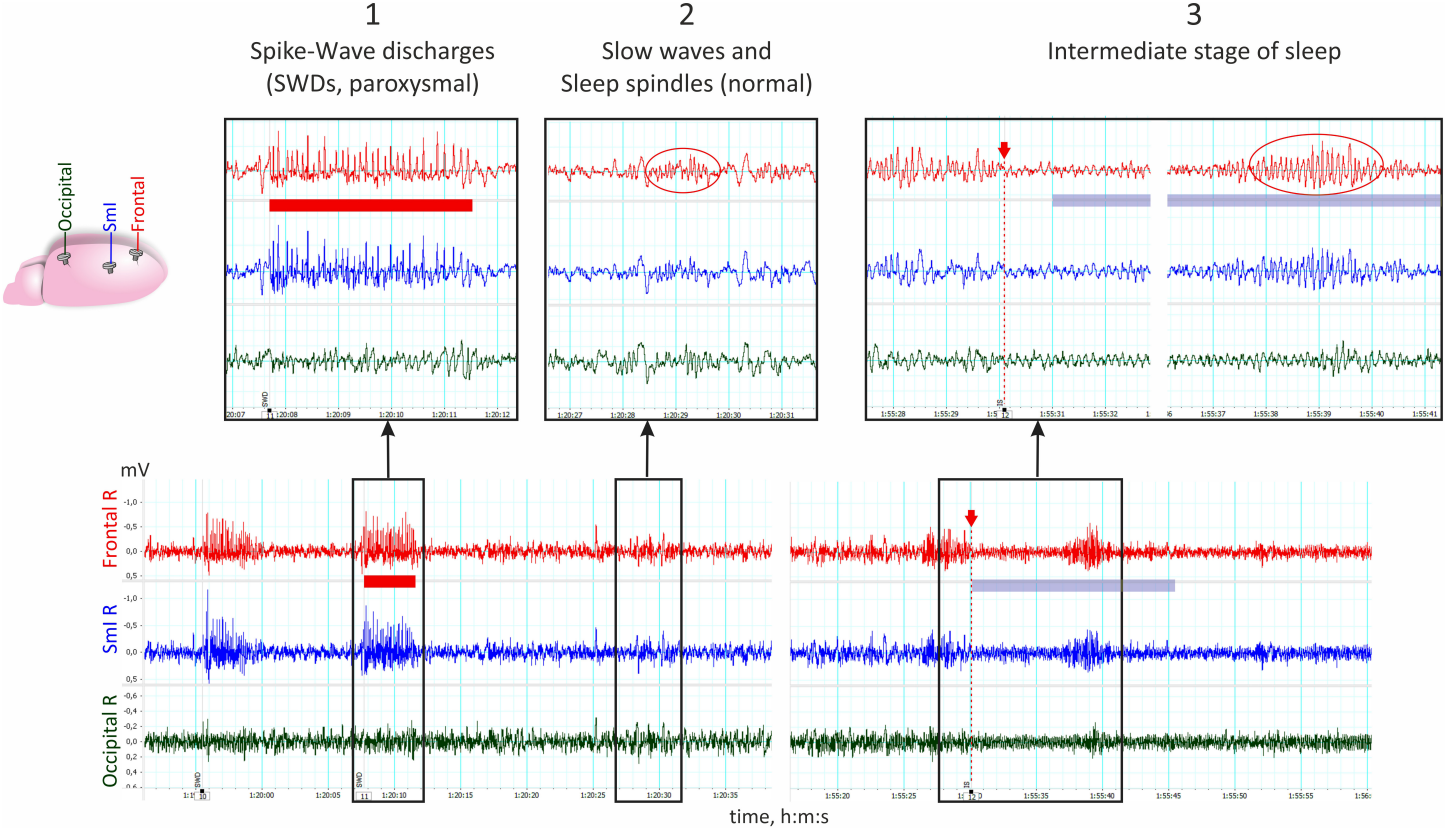
Electrical brain activity recorded with epidural electrodes in freely moving WAG/Rij rat. The schema of electrode placement is shown on the left. Electrode/miniscrews were implanted in right hemisphere over the frontal cortex (“Frontal R,” AP 2; L 2.5), somatosensory cortex (“SmI R,” AP −2; L 6), and occipital cortex (AP −5; L 4). All coordinates are given in mm relative to bregma, and the reference electrode was placed over the right cerebellum (not shown here). (1) Paroxysmal 8–10-Hz spike-wave discharges (SWDs) with the amplitude maximum in the anterior areas as marked with red stripe. (2) Physiological sleep spindles appeared as 10–14-Hz spindle-shaped waves in all cortical channels. (3) Intermediate state of sleep can be recognized at the end of NREM sleep (marked with blue stripe and red arrow indicating its onset) and characterized by the presence of occipital theta in conjunction with 6–8-Hz high-voltage anterior spindles (outlined by red oval).

In this perspective, I focus on two types of thalamocortical oscillations: sleep spindles and SWDs. Figure 1 demonstrates an example of electrocorticogram recorded in freely moving WAG/Rij rats, in which spontaneous 8–10-Hz SWDs (event 1) were followed by physiological sleep spindles (event 2). SWDs had a clearly paroxysmal profile with high-voltage spikes with amplitude maximum in the anterior areas (frontal and somatosensory cortex). Sleep spindles represented a brief sequence of 10–14-Hz spindle-shaped waves in anterior and posterior cortical areas (Figure 1, event 2—only frontal spindle was outlined by red oval).

Peculiarities of EEG sleep spindles in WAG/Rij rats were summed in the review (36). Sleep spindles in drug-naive WAG/Rij rats had lower intrinsic frequencies than those in non-epileptic Wistar rats at the age of 7 months (11.2 vs. 13.1 Hz) and at the age of 9 months (11.3 vs. 13.2 Hz) (37). Recently, we found out that the slow wave (~4 Hz) component immediately prior to normal sleep spindles in rats with spike-wave epilepsy was more powerful than that in non-epileptic control (38). In the same way, SWDs in WAG/Rij rats were preceded by delta precursor (with the mean frequency of 4.1 Hz in the frontal cortex) coexisting with theta (39). In addition to the abovementioned delta, cortical slow (<1 Hz) oscillations might play an important role in SWDs in WAG/Rij rats as far as it controls alternating active (Up) and silent (Down) cortical activity excitation (23, 40).

Electrographic pattern of SWDs in WAG/Rij rats is known to be immature before 2–3 months of age, and fully developed SWDs appeared at the age of 6 months. Relatively late onset of spike-wave epilepsy in WAG/Rij rats is beneficial in terms of the concept of genetically determined epileptogenesis (41). According to this concept, the pre-seizure period in young WAG/Rij rats represents the “latent period” of epileptogenesis, which is necessary for the development of spontaneous spike-wave seizures. The epileptogenic process in the thalamocortical circuitry causes SWDs in parallel to changes in sleep spindle EEG activity. This was recently confirmed by Kozák et al. (6) in Long Evans rats in which age-related dynamics of SWDs was similar to that in WAG/Rij rats (8–10). Age-related increase in SWDs in Long Evans rats correlated to a decrease in sleep spindle occurrence rate, suggesting “the possibility of mutually exclusive mechanisms of spindle and SWD generation” (6). Another important notion is that “maturation gradually shifts local ictal activity of the somatosensory cortex into global SWDs of the thalamocortical circuitry, which parallels the progressive disappearance of sleep spindles” (6). Developmental changes of sleep spindles in WAG/Rij rats between the age of 4 and 6 months were examined by van Luijtelaar and Bikbaev (42). They found no age-related changes in density of sleep spindles neither in anterior nor in posterior spindles.

The sleep process in WAG/Rij rats seems to be disrupted as it was indicated by several lines of evidence (28, 42, 43). Table 1 summarizes some of the more important findings obtained in male subjects.^1^ First, sleep structure. Six-month-old WAG/Rij rats, in which spike-wave seizures were fully developed, had a shorter sleep cycle when compared with 4-month-old WAG/Rij rats, in which seizures were immature, as measured in the morning and in the afternoon (42). Considering that sleep cycle in WAG/Rij rats was shorter than that in control inbred Black agouti ACI rats (42), spike-wave epilepsy results in shortening of sleep cycle. Second, fragmentation of slow-wave sleep in “epileptic” WAG/Rij rats (44). In 9–11-month-old WAG/Rij rats, we found negative correlations between the number of slow-wave sleep episodes and their mean duration, which was interpreted as sleep fragmentation (44).

**Table 1:** Literature data on sleep in WAG/Rij rats.

		**Animals**	**References**
Sleep cycle	Longer duration than in ACI rats	WAG/Rij vs. healthy control ACI^*^ rats (4–6 months old)	van Luijtelaar and Bikbaev (42)
	Shortening with age	WAG/Rij rats: age-related decrease in duration from 4 to 6 months of age (403 vs. 293 s) in parallel to 4.5-fold increase in number of SWDs	
NREM (non-rapid eye movement, slow-wave sleep)	Slow-wave sleep fragmentation	WAG/Rij: epileptic vs. non-epileptic subjects (9–11 months old)	Sitnikova et al. (44)
	Microarousals during slow-wave sleep	WAG/Rij: microarousals in epileptic subjects showed a tendency to be higher in number than in non-epileptic subjects (9 months old)	Runnova et al. (45)
IS stage^†^	Longer episode length than in Wistar rats	WAG/Rij vs. Wistar (adult age): 2.9 vs. 8.7 s	Gandolfo et al. (28)
	Transition IS stage → REM was less often than in Wistar Transition IS stage → arousal was more often than in Wistar Transition IS stage → NREM: more often than in Wistar	WAG/Rij vs. Wistar (adult age)	Gandolfo et al. (28)
	No age-related changes	WAG/Rij rats: the same number of IS episodes and IS duration at the age of 5–8 months in parallel to 6.6-fold increase in the total time spent in SWDs	Sitnikova et al. (11)
REM	Reduced number of episodes than in Wistar	WAG/Rij vs. Wistar (adult age)	Gandolfo et al. (28)

* *ACI, Black Agouti inbred rats*.

† *IS stage, intermediate sleep stage (33)*. *SWD, spike-wave discharge*.

## Microarousals and Spike-Wave Epilepsy

Arousals without awakening (microarousals) could be recognized as short intrusions of wakefulness into sleep. According to the American Academy of Sleep Medicine (47), microarousals are 3–15 s waking states preceded by at least 10 s of non-interrupted sleep. The nature of microarousals was thoroughly evaluated by the group of Péter Halász (48–50). Microarousals have attracted particular interest as markers of sleep dynamics and as phenomena related to the occurrence of interictal and ictal epileptic events during sleep (51). In fact, available standard sleep scoring systems reliably identify sleep states such as wake, REM, and non-rapid eye movement (NREM) sleep but do not identify short transient states, such as microarousals. Recently, Runnova et al. (45) introduced the new method for the automatic detection of microarousals in freely moving WAG/Rij rats using the abovementioned American Sleep Disorders Association (ASDA) criteria (47). They analyzed electrocorticographic recordings from frontal and occipital cortical areas during ~24 h. Microarousals were detected based on cumulative wavelet energy characteristics as measured in frontal and occipital channels in frequencies 5–10 Hz. It appeared that microarousals interrupted continuous sleep in all 16 WAG/Rij rats. In subjects with spike-wave epilepsy, the number of microarousals showed a strong tendency to be higher than that in subjects without spike-wave epilepsy. Therefore, spike-wave epilepsy in WAG/Rij rats seems to promote microarousals, suggesting putative associations between microarousals and spike-wave epilepsy. Further analysis of microarousals is needed in order to uncover these associations (i.e., temporal distribution of microarousals during sleep, analysis of their duration, and EEG structure). Consequently, both time–frequency and distribution analyses of microarousals and spike-wave discharges could be a key to better understanding the nature of sleep disturbances in spike-wave epilepsy.

## Intermediate Sleep Stage and Spike-Wave Epilepsy

The peculiar state, intermediate sleep stage (IS stage), could be recognized as a transitional state before and sometimes after REM sleep in humans, cats, and rats [reviewed in (52)]. IS state in rats “can be distinguished in EEG by the simultaneous presence of high-voltage spindle activity in the frontoparietal cortical areas and hippocampal theta activity” (53). Accordingly, the hallmarks of IS state in WAG/Rij rats are high-amplitude ~8-Hz frontal spindles and occipital theta (Figure 1). WAG/Rij rats displayed longer epochs of IS state than those in Wistar rats [Table 1; (28)]. The electroencephalographic profile of high-amplitude ~8-Hz frontal spindles during IS stage differed from 10 to 14-Hz sleep spindles and was similar to spike-wave spindles (SW spindles) described in (11). Spindles during the IS stage lasted 2–3 s, contained spikes or sharp waves (similar to SWD), and had waxing–winning morphology (similar to spindles). Interestingly, the number of episodes and duration of IS in WAG/Rij rats did not change with age from 5 to 8 months, but the total time spent in SWDs showed 6.6-fold increase during that period (11). Therefore, the IS state in WAG/Rij rats seems to be genetically pre-determined and may not be directly involved in epileptogenic processes within thalamocortical neuronal circuitry. In comparison with Wistar rats, WAG/Rij rats showed less transitions from IS stage to REM but more transitions from arousal and NREM sleep (28).

## Conclusion

Spike-wave epilepsy in WAG/Rij rat model of absence epilepsy is associated with mild sleep disturbances, such as slow-wave sleep fragmentation, a tendency to increase microarousals, enhanced intermediate stage of sleep, and reduced REM sleep.

## Author Contributions

The author confirms being the sole contributor of this work and has approved it for publication.

## Funding

This mini review was prepared within the state assignment of Ministry of Education and Science of the Russian Federation to Institute of Higher Nervous Activity for 2021–2023. This study was partly supported by the Russian Academy of Science and the Russian Foundation for Basic Research (Grant No. 19-015-00242).

## Conflict of Interest

The author declares that the research was conducted in the absence of any commercial or financial relationships that could be construed as a potential conflict of interest.

## Publisher's Note

All claims expressed in this article are solely those of the authors and do not necessarily represent those of their affiliated organizations, or those of the publisher, the editors and the reviewers. Any product that may be evaluated in this article, or claim that may be made by its manufacturer, is not guaranteed or endorsed by the publisher.

## Footnotes

^1^The group of Anton Coenen and Gilles van Luijtelaar did not find sex-related differences of SWDs in WAG/Rij rats (9, 10). Different results were published in 2021: male WAG/Rij rats showed higher mean duration and higher total duration of SWDs than females as measured at the age of 6, 7, 8, 9, and 10 months (46). It should be stressed that SWDs in female WAG/Rij rats are strongly modulated by ovarian hormone progesterone, and the number of SWDs in female subjects varied during the reproductive cycle (4–5 days). This might also affect sleep in female WAG/Rij rats; however, sex-related differences in sleep have not been systematically examined.
